# Whole-Genome Analysis of *Treponema pallidum* Subspecies *endemicum* among Men Who Have Sex with Men, Japan, 2020–2023

**DOI:** 10.3201/eid3204.251045

**Published:** 2026-04

**Authors:** Yuki Ohama, Kazuo Imai, Yuto Kotaka, Kenichi Lee, Ichiro Itoda, Shu-ichi Nakayama, Makoto Ohnishi, Yukihiro Akeda

**Affiliations:** National Institute of Infectious Diseases, Tokyo, Japan (Y. Ohama, Y. Kotaka, K. Lee, S. Nakayama, M. Ohnishi, Y. Akeda); Saitama Medical University, Saitama, Japan (K. Imai); Tokyo Metropolitan University, Tokyo (Y. Kotaka); Shirakaba Clinic, Tokyo (I. Itoda)

**Keywords:** Syphilis, Treponema pallidum, Treponema pallidum subsp. endemicum, bacteria, whole-genome sequencing, men who have sex with men, sexually transmitted infections, STDs, Japan

## Abstract

Whole-genome sequencing of *Treponema pallidum* subsp. *endemicum* strains from men who have sex with men in Japan revealed a genetically distinct lineage from other geographic regions circulating via sexual transmission. Strengthening global molecular epidemiologic surveillance is essential for clarifying epidemiologic trends, clinical characteristics, and transmission pathways of this subspecies.

*Treponema pallidum* bacteria comprise 3 distinct subspecies: *T. pallidum* subsp. *pallidum* (TPA), *T. pallidum* subsp. *pertenue*, and *T. pallidum* subsp. *endemicum* (TEN) (*1*). TPA causes syphilis, a sexually transmitted infection prevalent worldwide. TEN causes bejel, which primarily affects children, and is transmitted through close contact (*1*). TEN is endemic to arid regions (e.g., Middle East and parts of Africa), particularly in populations with low socioeconomic status (*2*). Recent molecular studies revealed TEN in adults initially suspected of having syphilis (*3**-**7*). Evidence suggests that TEN can be transmitted sexually in nonendemic regions, including France, Cuba, and Japan (*3**-**5*). In Japan, researchers first identified a TEN infection in 2014, and 13 cases have since been reported among men who have sex with men (MSM) in urban centers, including Yamaguchi, Osaka, Kyoto, Hyogo, and Tokyo (*6**,**7*). Although typically considered nonvenereal, confirmed TEN have recently surfaced in MSM in nonendemic countries, raising questions about the bacterium’s transmission dynamics and potential for sexual spread.

The National Institute of Infectious Diseases (Tokyo, Japan) serves as a reference laboratory and conducts surveillance of syphilis cases and molecular epidemiologic surveillance of TPA. That surveillance identified 5 new cases of TEN during 2020–2023. Initially, we performed multilocus sequence typing (MLST) to investigate circulating TPA sequence types and identify TEN in Japan. We followed that investigation by applying whole-genome sequencing (WGS) and single nucleotide polymorphism–based comparative analysis to assess phylogeny and possible sexual transmission of TEN in nonepidemic regions.

## The Study

For our study, we obtained specimens through multicenter syphilis surveillance in Tokyo and Osaka during 2020–2023 (Appendix). Four clinics from 2 prefectures participated. The case definition included adult patients >18 years of age suspected of having primary or secondary syphilis. Submitted specimens were from 97 male patients, 98 female patients, and 2 persons of unknown sex. We performed PCR testing on collected specimens, targeting *TpN47* and *pol*A genes. We subjected residual samples to molecular epidemiologic analysis of TPA, using MLST and a microbial genome diversity database (PubMLST, https://pubmlst.org) in analyzing 3 genes: *TP0136*, *TP0548*, and *TP0705*. 

We collected all 197 specimens from symptomatic sites, including swab samples from genital, anal, and oral lesions. Among the 97 male patients, we identified 28 (28.9%) as MSM. Using PubMLST, we detected TEN in 5 male patients who visited sexually transmitted infection clinics in Tokyo and Osaka. We identified 4 of those patients as MSM, noting 1 man did not disclose his sexual orientation (Table). Clinic staff noted genital induration, ulcers, or erosions in all 5 patients, findings similar to those of syphilis. In addition, testing revealed positive results for both rapid plasma reagin and *T. pallidum* antibodies, and PCR targeting the *TpN47* and *pol*A genes also brought positive results. Based on the data, physicians determined a diagnosis of syphilis for all 5 patients. Further analysis included WGS (Appendix), which rendered high-quality TEN genomes from 3 strains isolated in Tokyo with >90% reference coverage and <5% estimated contamination (GenBank accession nos. DRX678426–8). We then conducted phylogenetic analysis using 8 available TEN genomes from GenBank (https://www.ncbi.nlm.nih.gov/genbank) (Table).

**Table:** Summary of strains from a whole-genome analysis of *Treponema pallidum* subspecies *endemicum* among men who have sex with men, Japan, 2020–2023*

Strain name	Year detected	Patient sex	Sexual orientation	Source of transmission	Location	GenBank accession no.	Reference
NIIDTP373	2020	M	MSM	Sexual	Osaka, Japan	Not available	This study
NIIDTP389	2020	M	MSM	Sexual	Tokyo, Japan	Not available	This study
NIIDTP404	2021	M	MSM	Sexual	Tokyo, Japan	DRX678426	This study
NIIDTP415	2021	M	MSM	Sexual	Tokyo, Japan	DRX678427	This study
NIIDTP523	2022	M	Unknown	Sexual	Tokyo, Japan	DRX678428	This study
Japan320e	2019	M	MSM	Sexual	Tokyo, Japan	NZ_CP073523	(*7*)
Japan322e	2019	M	MSM	Sexual	Tokyo, Japan	NZ_CP073522	(*7*)
Japan326e	2019	M	MSM	Sexual	Tokyo, Japan	NZ_CP073518	(*7*)
Japan346e	2019	M	Unknown	Sexual	Tokyo, Japan	NZ_CP073506	(*7*)
Iraq B	1951	F	Unknown	Unknown	Iraq	NZ_CP032303	(*8*)
Bosnia A	1950	M	Unknown	Unknown	Bosnia	NZ_CP007548	(*9*)
C77	2014	M	MSM	Sexual	Cuba	NZ_CP081507	(*10*)
C279	2017	M	MSM	Sexual	Cuba	NZ_CP078090	(*10*)

*MSM, men who have sex with men.

To accurately assess genetic relatedness, we analyzed aligned core genes and constructed a maximum-likelihood phylogenetic tree (Figure). That analysis showed that 7 TEN strains from Japan, isolated from 5 MSM and 2 male patients with unknown sexual orientation in Tokyo during 2019–2022 (*7*), belonged to the same clade, distinct from TEN strains from endemic regions, including Iraq B (1951) (*8*), Bosnia A (1950) (*9*), and Cuba (2 strains from MSM, 2014 and 2017) (*10*).

**Figure:**
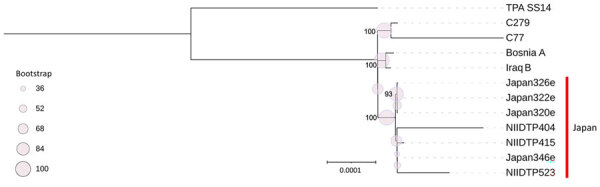
Phylogenetic analysis of isolates from a study of whole-genome analysis of *Treponema pallidum* subspecies *endemicum* among men who have sex with men, Japan, 2020–2023. Open reading frames annotated from de novo assembled contigs using Bakta version 1.7.0 (https://bakta.computational.bio), and corresponding GFF3 annotation files processed with Panaroo version 1.2.3 (https://zenodo.org/records/3945497) to define core genomes across isolates. Multiple alignments of core gene sequences generated by Panaroo were used for phylogenetic reconstruction. *T. pallidum* subsp. *pallidum* SS14 (GenBank accession no. NC_021508) was used as a reference strain. IQ-TREE version 2.2.0 (http://www.iqtree.org) was used to infer maximum-likelihood phylogenies, incorporating 1,000 ultrafast bootstrap replicates to assess nodal support. Phylogenetic tree was visualized by using Interactive Tree of Life (iTOL; https://itol.embl.de). The *T. pallidum* subsp. *endemicum* strains isolated in Japan formed a clade distinct from classic endemic strains, including Iraq B and strains from Cuba (C77 and C279). Scale bar indicates nucleotide substitutions per site.

## Conclusion

Phylogenetic analysis based on WGS of TEN isolates from 8 male patients (7 MSM and 1 of unknown sexual orientation) with syphilitic symptoms in 2019–2022 revealed that all Tokyo strains belonged to a single clade. That finding provides strong molecular evidence of sustained, local transmission of TEN among MSM in Tokyo.

Clustering of genetically near-identical TEN strains, isolated over several years from epidemiologically linked populations, supports an ongoing transmission network. Given that *T. pallidum* has a highly conserved genome with low single-nucleotide polymorphism accumulation rates, this distinct clade among these Japan isolates further substantiates their shared origin and recent divergence. Since 2019, reports of sporadic cases of TEN in Tokyo have continued to surface. In an MLST analysis, researchers identified TEN in 3 of 26 patients with HIV identifying as MSM who presented with syphilitic symptoms at Tokyo hospitals during 2019–2022 (*11*). Similar research revealed 1 case of TEN on the basis of both MLST and multilocus sequence analysis (MLSA) among 48 patients given a diagnosis of syphilis in Tokyo during 2023–2024 (*12*). These findings support the persistent circulation of TEN as a sexually transmitted infection among MSM in Tokyo.

In addition to the Tokyo cases, research uncovered 5 other cases of TEN infection during 2014–2018 among MSM in western Japan, including Yamaguchi, Osaka, Kyoto, and Hyogo prefectures (*6*). However, in those earlier investigations, clinicians analyzed isolates only by MLSA targeting *TP0548* and *TP0856* and we thus excluded that data from our study’s phylogenetic analysis because of the lack of WGS data. In our study, testing resulted in TEN detection in 2 patients identifying as MSM; however, we did not achieve high-quality genome sequencing. Therefore, whether the strains from outside the core Tokyo and Kansai cohort belong to the same transmission cluster remains unclear. Nevertheless, our results underscore the potential undetected circulation of sexually transmitted TEN in nonendemic regions, highlighting the critical role of molecular surveillance in uncovering hidden transmission patterns. The MLSA results indicated that these earlier strains also formed a distinct clade from Iraq B and Bosnia A, supporting the conclusion that TEN strains in Japan are genetically distinct. Furthermore, research has yet to isolate TEN from female patients.

Clinically, distinguishing between TPA and sexually transmitted TEN is challenging. Previous reports indicate that sexually transmitted TEN imparts symptoms indistinguishable from syphilis caused by TPA, and the medical literature offers no unique clinical markers for TEN infection. Serologic tests yield similar results for both subspecies. In addition, molecular targets like *TpN47* and *pol*A, commonly used in primary syphilis diagnostics, are present in both TPA and TEN. Therefore, only WGS, MLST, MLSA, or specific genetic assays can differentiate TPA from TEN (*13*,*14*). Accordingly, many TEN infections in Japan could be misclassified as syphilis caused by TPA. In our study, all 5 patients confirmed to have TEN received an initial diagnosis of typical syphilis.

Since 2012, reported syphilis cases in Japan have increased >10-fold, particularly among MSM in urban centers such as Tokyo, indicating expanding sexual transmission networks (Japan Institute for Health Security, https://id-info.jihs.go.jp/niid/en/iasr/9542-479te.html). Within that context, repeated detection of genetically related TEN strains suggests this typically nonvenereal pathogen could be established in these networks. Those findings suggest that TEN might be underdiagnosed and more widespread than currently recognized. In addition, TEN might have begun to spread beyond MSM populations through sexual transmission, raising the possibility of broader outbreaks. 

In summary, although prior studies have suggested that TEN can be transmitted through close oral contact, such as kissing (*2*), the exact transmission route in the cases reported here remains unclear. To clarify epidemiologic trends, clinical characteristics, and transmission pathways of TEN, strengthening of molecular surveillance and continuing to accumulate clinical and genomic data are essential.

Appendix Additional information for whole-genome analysis of *Treponema pallidum* subsp. *endemicum* among men who have sex with men, Japan, 2020–2023
