# Supplementary material for: Whole-Genome Analysis of Treponema pallidum Subspecies endemicum among Men Who Have Sex with Men, Japan, 2020–2023
Source: Emerg Infect Dis. 2026 Apr;32(4):636–9. doi: 10.3201/eid3204.251045 (PMC13094845; doi:10.3201/eid3204.251045)
Supplement: Appendix — Additional information for whole-genome analysis of Treponema pallidum subsp. endemicum among men who have sex with men, Japan, 2020–2023 [file 25-1045-Techapp-s1.pdf]

Article DOI: https://doi.org/10.3201/eid3204.251045

EID cannot ensure accessibility for supplementary materials supplied by authors. Readers who have difficulty accessing supplementary content should contact the authors for assistance.

Whole-Genome Analysis of *Treponema pallidum* subsp. *endemicum* among Men Who Have Sex with Men, Japan, 2020–2023

Appendix

Materials and Methods

Clinical Samples and DNA extraction

A multicenter surveillance study to collect clinical specimens from patients suspected of having syphilis was conducted in Tokyo and Osaka, Japan, from 2020 through 2023. Three clinics in Tokyo and one clinic in Osaka participated in the surveillance and provided specimens in this study (Appendix Figure). Specimens were collected according to the following criteria: (i) adults aged 18 years or older and (ii) patients with symptomatic primary or secondary syphilis from whom samples could be obtained for syphilis diagnosis and molecular surveillance. For specimen submission, swab samples were obtained from genital, anal, or oral lesions. The submitted swabs were suspended in TE buffer, and DNA was extracted. Because the anatomic site of specimen collection was not recorded for individual samples, detailed information on the specific collection sites was unavailable. *Treponema pallidum* DNA was detected using polymerase chain reaction (PCR) assays targeting the highly conserved *TpN47* (*1*) and *polA* (*2*)

genes. Samples yielding amplification of either target were considered positive. Basic demographic information, including sex and self-reported sexual orientation, was collected through medical records. No other clinical metadata were systematically recorded.

Molecular Typing and MLST Analysis

Positive samples were subjected to Multilocus Sequence Typing (MLST) using PCR amplification and Sanger sequencing of three *T. pallidum* loci: *TP0136*, *TP0548*, and *T P0705* (*3*). Sequences were submitted to the *Treponema pallidum* MLST database (PubMLST BIGSdb; https://pubmlst.org/tpallidum/) to determine allele profiles and assign sequence types.

Whole-Genome Sequencing (WGS), Assembly, and Quality Assessment

Genomic DNA was extracted using a DNeasy Blood & Tissue Kit (Qiagen, https://www.qiagen.com). Whole-genome amplification was performed using a GenomePhi V2 DNA Amplification Kit (GE Healthcare, https://www.gehealthcare.com) according to the manufacturer's instructions. Libraries were constructed using the SureSelect QXT Target Enrichment System (Agilent Technologies, https://www.agilent.com), targeting the Nichols strain genome via custom capture probes, and sequenced on an Illumina MiSeq platform (2 × 300 bp paired-end reads) (Illumina, https://www.illumina.com).

Raw sequencing reads were quality-filtered using Fastq v0.23.4 (*4*) to remove adaptors and low-quality bases. Reads were mapped to three reference genomes:

(a) *T. pallidum* subsp. *pallidum* strain Nichols (GenBank Accession no: NC_021490.2)

(b) *T. pallidum* subsp. *pallidum* strain SS14 (GenBank Accession no: CP004011.1)

(c) *T. pallidum* subsp. *endemicum* strain Iraq B (GenBank Accession no: CP032303.1)

Reads were aligned using Bowtie2 v2.5.4 (*5*), and the mapped reads were extracted and assembled using SPAdes v4.0.0 (*6*) with default parameters. The quality of assemblies, including completeness and contamination, was assessed using CheckM v1.2.2 (*7*). Only high-quality genomes with >90% reference coverage and <5% estimated contamination were retained for downstream analyses.

Single Nucleotide Polymorphism (SNP) Calling and Global Phylogenetic Analysis

Due to the extremely low level of sequence variation and insufficient (SNPs) in *T. pallidum*, core-genome SNP calling using two independent pipelines did not yield sufficient resolution to reconstruct a robust phylogeny. As an alternative approach, phylogenetic inference was conducted using core-gene alignments derived from de novo assembled contigs. Open reading frames were annotated using Bakta v1.7.0 (*8*), and core gene clusters were identified using Panaroo v1.2.3 (*9*) based on the generated GFF3 files. Multiple sequence alignments of conserved genes across all isolates were computed within the Panaroo framework. The resulting core gene alignment was used to infer a maximum-likelihood phylogeny with IQ-TREE v2.2.0 (*10*), employing the best-fit substitution model selected via Bayesian information criterion (BIC). Node support was assessed with 1,000 ultrafast bootstrap replicates. The final tree was visualized using Interactive Tree of Life (iTOL) (https://itol.embl.de/).

References

1. Orle KA, Gates CA, Martin DH, Body BA, Weiss JB. Simultaneous PCR detection of *Haemophilus ducreyi, Treponema pallidum*, and herpes simplex virus types 1 and 2 from genital ulcers. J Clin Microbiol. 1996;34:49–54. PubMed https://doi.org/10.1128/jcm.34.1.49-54.1996

2. Liu H, Rodes B, Chen CY, Steiner B. New tests for syphilis: rational design of a PCR method for detection of *Treponema pallidum* in clinical specimens using unique regions of the DNA polymerase I gene. J Clin Microbiol. 2001;39:1941–6. PubMed https://doi.org/10.1128/JCM.39.5.1941-1946.2001

3. Grillová L, Bawa T, Mikalová L, Gayet-Ageron A, Nieselt K, Strouhal M, et al. Molecular characterization of *Treponema pallidum* subsp. *pallidum* in Switzerland and France with a new multilocus sequence typing scheme. PLoS One. 2018;13:e0200773. PubMed https://doi.org/10.1371/journal.pone.0200773

4. Chen S. Ultrafast one-pass FASTQ data preprocessing, quality control, and deduplication using fastp. iMeta. 2023;2:e107. PubMed https://doi.org/10.1002/imt2.107

5. Li H, Durbin R. Fast and accurate long-read alignment with Burrows-Wheeler transform. Bioinformatics. 2010;26:589–95. PubMed https://doi.org/10.1093/bioinformatics/btp698

6. Bankevich A, Nurk S, Antipov D, Gurevich AA, Dvorkin M, Kulikov AS, et al. SPAdes: a new genome assembly algorithm and its applications to single-cell sequencing. J Comput Biol. 2012;19:455–77. PubMed https://doi.org/10.1089/cmb.2012.0021

7. Parks DH, Imelfort M, Skennerton CT, Hugenholtz P, Tyson GW. CheckM: assessing the quality of microbial genomes recovered from isolates, single cells, and metagenomes. Genome Res. 2015;25:1043–55. PubMed https://doi.org/10.1101/gr.186072.114

8. Schwengers O, Jelonek L, Dieckmann MA, Beyvers S, Blom J, Goesmann A. Bakta: rapid and standardized annotation of bacterial genomes via alignment-free sequence identification. Microb Genom. 2021;7:000685. PubMed https://doi.org/10.1099/mgen.0.000685

9. Tonkin-Hill G, MacAlasdair N, Ruis C, Weimann A, Horesh G, Lees JA, et al. Producing polished prokaryotic pangenomes with the Panaroo pipeline. Genome Biol. 2020;21:180. PubMed https://doi.org/10.1186/s13059-020-02090-4

10. Nguyen LT, Schmidt HA, von Haeseler A, Minh BQ. IQ-TREE: a fast and effective stochastic algorithm for estimating maximum-likelihood phylogenies. Mol Biol Evol. 2015;32:268–74. PubMed https://doi.org/10.1093/molbev/msu300

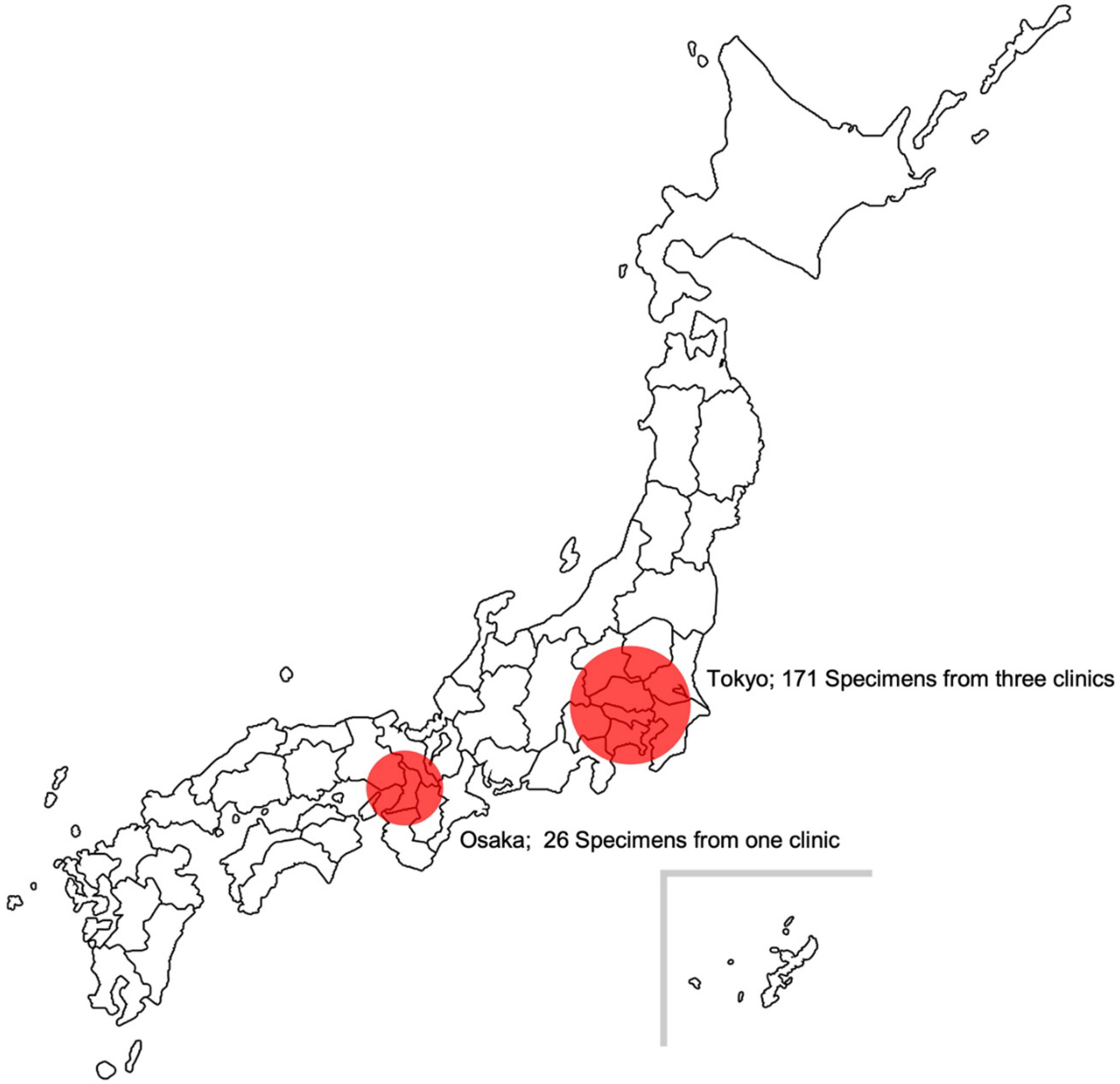

Appendix Figure. Distribution of specimen submissions for a study of whole-genome analysis of *Treponema pallidum* subsp. *endemicum* among men who have sex with men, Japan, 2020–2023.
